# Early (Days 1–4) post-treatment serum hCG level changes predict single-dose methotrexate treatment success in tubal ectopic pregnancy

**DOI:** 10.1093/humrep/dead089

**Published:** 2023-05-13

**Authors:** Scott C Mackenzie, Catherine A Moakes, Ann M Doust, Ben W Mol, W Colin Duncan, Stephen Tong, Andrew W Horne, Lucy H R Whitaker

**Affiliations:** MRC Centre for Reproductive Health, University of Edinburgh, Edinburgh, UK; Birmingham Clinical Trials Unit, University of Birmingham, Birmingham, UK; MRC Centre for Reproductive Health, University of Edinburgh, Edinburgh, UK; Department of Obstetrics and Gynaecology, Monash University, Melbourne, Australia; Aberdeen Centre for Women's Health Research, School of Medicine, Medical Sciences and Nutrition, University of Aberdeen, Aberdeen, UK; MRC Centre for Reproductive Health, University of Edinburgh, Edinburgh, UK; Department of Obstetrics and Gynaecology, University of Melbourne, Melbourne, Australia; MRC Centre for Reproductive Health, University of Edinburgh, Edinburgh, UK; MRC Centre for Reproductive Health, University of Edinburgh, Edinburgh, UK

**Keywords:** tubal ectopic pregnancy, methotrexate, human chorionic gonadotropin, biomarker, treatment success

## Abstract

**STUDY QUESTION:**

What is the capacity of the change between Day 1 and Day 4 post-treatment serum human chorionic gonadotropin (hCG) levels for predicting single-dose methotrexate treatment success in tubal ectopic pregnancy?

**SUMMARY ANSWER:**

Any fall in Days 1–4 serum hCG signified an 85% (95% CI 76.8–90.6) likelihood of treatment success for women with tubal ectopic pregnancy (initial hCG of ≥1000 and ≤5000 IU/l) managed with single-dose methotrexate.

**WHAT IS KNOWN ALREADY:**

For those with tubal ectopic pregnancy managed by single-dose methotrexate, current guidelines advocate intervention if Days 4–7 hCG fails to fall by >15%. The trajectory of hCG over Days 1–4 has been proposed as an early indicator that predicts treatment success, allowing early reassurance for women. However, almost all prior studies of Days 1–4 hCG changes have been retrospective.

**STUDY DESIGN, SIZE, DURATION:**

This was a prospective cohort study of women with tubal ectopic pregnancy (pre-treatment hCG of ≥1000 and ≤5000 IU/l) managed with single-dose methotrexate. The data were derived from a UK multicentre randomized controlled trial of methotrexate and gefitinib versus methotrexate and placebo for treatment of tubal ectopic pregnancy (GEM3). For this analysis, we include data from both treatment arms.

**PARTICIPANTS/MATERIALS, SETTING, METHODS:**

Participants were categorized according to single-dose methotrexate treatment success or failure. Treatment success for this analysis was defined as complete and uneventful resolution of tubal ectopic pregnancy to serum hCG <30 IU/l following single-dose methotrexate treatment without additional treatment. Patient characteristics of the treatment success and failure groups were compared. Changes in Days 1–4, 1–7, and 4–7 serum hCG were evaluated as predictors of treatment success through receiver operating characteristic curve analysis. Test performance characteristics were calculated for percentage change ranges and thresholds including optimal classification thresholds.

**MAIN RESULTS AND THE ROLE OF CHANCE:**

A total of 322 women with tubal ectopic pregnancy were treated with single-dose methotrexate. The overall single-dose methotrexate treatment success rate was 59% (n = 189/322). For any fall in serum hCG on Days 1–4, likelihood ratios were >3, while for any fall of serum hCG >20% on Days 1–7, likelihood ratios reached 5. Any rise of serum hCG on Days 1–7 and 4–7 strongly reduced the chance of success. Any fall in Days 1–4 hCG predicted single-dose methotrexate treatment success with a sensitivity of 58% and specificity 84%, resulting in positive and negative predictive values of 85% and 57%, respectively. Any rise in Days 1–4 serum hCG <18% was identified as an optimal test threshold that predicted treatment success with 79% sensitivity and 74% specificity, resulting in 82% positive predictive value and 69% negative predictive value.

**LIMITATIONS, REASONS FOR CAUTION:**

Our findings may be limited by intervention bias resulting from existing guidelines which influences evaluation of hCG changes reliant on Day 7 serum hCG levels.

**WIDER IMPLICATIONS OF THE FINDINGS:**

Examining a large prospective cohort, we show the value of Days 1–4 serum hCG changes in predicting single-dose methotrexate treatment success in tubal ectopic pregnancy. We recommend that clinicians provide early reassurance to women who have a fall or only a modest (<18%) rise in Days 1–4 serum hCG levels, that their treatment will likely be effective.

**STUDY FUNDING/COMPETING INTEREST(S):**

This project was supported by funding from the Efficacy and Mechanism Evaluation programme, a Medical Research Council and National Institute for Health Research partnership (grant reference number 14/150/03). A.W.H. has received honoraria for consultancy for Ferring, Roche, Nordic Pharma and AbbVie. W.C.D. has received honoraria from Merck and Guerbet and research funding from Galvani Biosciences. L.H.R.W. has received research funding from Roche Diagnostics. B.W.M. is supported by a NHMRC Investigator grant (GNT1176437). B.W.M. also reports consultancy for ObsEva and Merck and travel support from Merck. The other authors declare no competing interests.

**TRIAL REGISTRATION NUMBER:**

This study is a secondary analysis of the GEM3 trial (ISRCTN Registry ISRCTN67795930).

## Introduction

Ectopic pregnancies are a leading cause of first trimester maternal morbidity and mortality and complicate approximately 2% of all pregnancies (Marion and Meeks, 2012; Taran *et al.*, 2015). Although an ectopic pregnancy can resolve spontaneously (Korhonen *et al.*, 1994), tubal rupture and associated haemorrhage requiring emergency surgery remains a risk (Westaby *et al.*, 2012). When the diagnosis precedes tubal rupture, management can include surgical, medical or expectant approaches. Around 25–30% of those presenting with ectopic pregnancy are eligible for medical management (Jurkovic and Wilkinson, 2011).

Methotrexate, an anti-folate cytotoxic drug, is an established, safe medical treatment for unruptured ectopic pregnancy that allows many haemodynamically stable women to avoid surgery. However, 29% of those treated with single-dose methotrexate will experience treatment failure, requiring rescue surgery (Horne *et al.*, 2023). Through serial monitoring of post-methotrexate serum human chorionic gonadotropin (hCG) levels, identification of individuals likely to experience treatment success or failure is possible.

Current UK guidelines advise that serum hCG is measured on Day 4 and Day 7 post-methotrexate treatment because a >15% reduction in hCG levels in this period indicates likely treatment success. Patients with ≤15% reduction in hCG levels are considered for further methotrexate treatment and/or surgery (Elson *et al.*, 2016; American College of Obstetricians and Gynecologists, 2018). This ‘>15% rule’ originates from the original single-dose methotrexate treatment protocol proposed by Stovall *et al.* (1991). Although justification for its use was not provided when introduced, the ‘>15% rule’ has prevailed in the literature and clinically since. It was subsequently validated in 2007 by Kirk *et al.* (2007) where it was found to have a positive predictive value (PPV) of 93% for single-dose methotrexate treatment success.

Stovall’s protocol does not predict response earlier than 7 days post-methotrexate treatment. The advantages of even earlier prognostic information are numerous: earlier identification of likely methotrexate treatment failure allows for the option of earlier surgical intervention, which would then reduce the risk of rupture. Earlier prognostic information also provides earlier reassurance and reduces the follow-up burden for those involved in what can be a distressing and uncertain time (Farren *et al.*, 2016). These benefits have led groups in recent years to investigate the predictive value of trends associated with Day 4 serum hCG levels, namely percentage changes between Day 1 and Day 4 hCG (Agostini *et al.*, 2007; Nguyen *et al.*, 2010; Skubisz *et al.*, 2011, 2013; Ustunyurt *et al.*, 2013; Levin *et al.*, 2018; Mashiach et al., 2018; Wong *et al.*, 2018; Levin *et al.*, 2019; Goh *et al.*, 2020; Zhang *et al.*, 2020). These studies, of which all but one of which are retrospective in nature, have investigated a variety of metrics using pre-treatment or Day 4 hCG that are predictive of treatment outcome, with varying degrees of utility.

Utilizing prospectively collected data from a large UK ectopic pregnancy clinical trial cohort (Horne *et al.*, 2023), we aimed to determine whether the change in Day 1 and Day 4 post-treatment serum hCG levels could predict single-dose methotrexate treatment success.

## Materials and methods

### Ethical approval

This study is a secondary analysis of data from a multicentre, randomized, double-blind, placebo-controlled trial of combination of gefitinib and methotrexate to treat tubal ectopic pregnancy (GEM3) (Horne *et al.*, 2023). Ethical approval for the trial was obtained from the Scotland A Research Ethics Committee (REC 16/SS/0014). All trial participants provided written informed consent. The trial protocol has been published (May *et al.*, 2018).

### Participants

The GEM3 trial inclusion and exclusion criteria are published in full elsewhere (May *et al.*, 2018; Horne *et al.*, 2023). In summary, the trial participants studied in this secondary analysis of the GEM3 data were aged 18–50 years, diagnosed with definite or probable (adenexal mass on ultrasound with sub-optimal serial hCG) tubal ectopic pregnancy, who were haemodynamically stable, with pre-treatment serum hCG concentrations ≥1000 and ≤5000 IU/l, in whom a clinical decision had been made for medical management with methotrexate. Participants were administered a single-dose intramuscular injection of methotrexate (50 mg/m^2^) and randomized to 7 days of either oral gefitinib or identical placebo. Serum hCG monitoring followed standard clinical practice with hCG typically sampled on Day 1 (day of methotrexate), Day 4, Day 7 then weekly until ectopic pregnancy resolution. The primary outcome of the GEM3 trial was surgical intervention for treatment of the index ectopic pregnancy.

### Outcomes

Treatment success for this analysis was defined as complete and uneventful resolution of tubal ectopic pregnancy (serum hCG <30 IU/l) following single-dose methotrexate treatment without additional treatment. Participants who required further treatment, medical (further methotrexate) or surgical (salpingectomy or salpingostomy by laparoscopy or laparotomy) were considered to have failed single-dose methotrexate treatment. The decision for surgical intervention was at the discretion of the responsible clinician and the rationale for this decision may have included worsening clinical symptoms, increasing serum hCG levels, clinical and/or laboratory and/or ultrasound signs of intra-abdominal bleeding, and participant’s request for surgery.

Participants were categorized into treatment success or failure groups as defined above. We assessed the percentage change in serum hCG between Day 1 and Day 4, Day 1 and Day 7, and Day 4 and Day 7, where Day 1 was the date of methotrexate treatment.

Longstanding variability is present in post-methotrexate serum hCG data resulting from inconsistency in defining the day of methotrexate administration as Day 0 or Day 1. Consequently, those who define the day of methotrexate as Day 0 will perform venepuncture for subsequent monitoring hCGs a day later than those who define the day of methotrexate as Day 1. To account for this variability, to reflect current clinical practice and to minimize data missingness, we extended our definition of Day 4 to include Days 3 and 4, of Day 7 to include Days 6 and 7 and of Days 4–7 to include Days 3–6. We adopted Day 1 as the day of methotrexate administration as our data showed clinicians most commonly used this definition based on the timing of subsequent blood sampling.

### Statistical analysis

We compared baseline characteristics of participants with treatment success versus those with treatment failure. Normally and non-normally distributed continuous variables were analysed using a Student’s *t*-test and Wilcoxon rank-sum test, respectively. Categorical variables were analysed using a Fisher’s exact (number of previous presumed ectopic pregnancies/pregnancies of unknown location, number of presumed patent tubes, current IVF pregnancy) or Chi-squared test (ethnicity, smoking status, previous live births, previous chlamydia infection, ectopic pregnancy size).

Receiver operating characteristic (ROC) curve analysis was performed to evaluate percent in Days 1–4, 1–7, and 4–7 serum hCG change, and area under curve (AUC) for each marker was calculated alongside an optimal classification threshold, identified through maximalization of Youden’s index (sensitvity+specificity-1). Test performance characteristics including sensitivity, specificity, PPV, negative predictive value (NPV), likelihood ratio of a positive test and likelihood ratio of a negative test were calculated with associated exact 95% CIs, for the optimal thresholds alongside previously investigated thresholds including any fall in hCG, a >15% fall and a >20% fall within each time interval assessed.

We included all women if they received either gefitinib or placebo in combination with methotrexate since there was evidence of ineffectiveness of gefitinib versus placebo in addition to methotrexate (Horne *et al.*, 2023). However, to support this decision, subgroup analyses were performed comparing the AUC for methotrexate/gefitinib, and methotrexate/placebo groups for each hCG interval using easyROC v1.3.1 (Goksuluk *et al.*, 2016) where AUC standard error and CI were calculated using DeLong *et al.* (1988)’s estimate.

A *P*-value <0.05 was considered to indicate statistical significance. Statistical analysis was performed with R v4.1.1 using *epiR* (Stevenson *et al.*, 2022) and *ROCR* v1.0-11 (Sing *et al.*, 2005).

## Results

Participants (n = 328) were randomly assigned to methotrexate/placebo (n = 163) and methotrexate/gefitinib (n = 165) groups. Both randomized groups had similar participant characteristics at study enrolment (Horne *et al.*, 2023).

Following randomization, three participants withdrew, one had hCG <1000 IU/l prior to methotrexate treatment and two had surgery prior to methotrexate treatment. Thus, 322 women received their initial dose of methotrexate and were followed up until resolution of pregnancy.

For the analysis, most participant characteristics including risk factors for ectopic pregnancy (age, smoking status, prior chlamydia infection and IVF treatment) were similar among single-dose methotrexate treatment success and failure groups. Day 1 and Day 4 serum hCG were both significantly lower in the treatment success groups (see Table 1).

**Table 1.** Participant characteristics for methotrexate treatment success and failure groups.

	Single-dose methotrexate treatment success (n = 189)	Single-dose methotrexate treatment failure (n = 133)	*P*
Age (years)	31.6 (5.3)	31.8 (5.6)	0.70
BMI (kg/m^2^)	26.5 (5.8)	27.4 (6.5)	0.19
Ethnicity, n (%)			
White	143 (76)	101 (76)	0.16
Asian	28 (15)	13 (10)	
Black	8 (4)	13 (10)	
Other	8 (4)	6 (5)	
Missing/not stated	2	0	
Smoking status, n (%)			
Current smoker	47 (26)	26 (20)	0.51
Ex-smoker	34 (19)	24 (19)	
Never smoked	101 (55)	78 (61)	
Unknown	7	5	
Previous live births, n (%)			
0	104 (55)	70 (53)	0.85
1	46 (24)	37 (28)	
2	26 (14)	16 (12)	
≥3	12 (6)	10 (8)	
Unknown	1	0	
Current IVF pregnancy, n (%)			
Yes	3 (2)	4 (3)	0.45
No	185 (98)	128 (97)	
Unknown	1	1	
Previous chlamydia infection, n (%)			
Yes	26 (16)	20 (17)	1
No	136 (84)	100 (83)	
Unknown	27	13	
Number of presumed patent tubes, n (%)			
0	0 (–)	1 (1)	0.66
1	28 (15)	20 (15)	
2	160 (85)	112 (84)	
Missing	1	0	
Number of previous presumed ectopic pregnancies/pregnancies of unknown location, n (%)			
0	157 (84)	110 (83)	0.84
1	23 (12)	19 (14)	
2	7 (4)	3 (2)	
≥3	1 (1)	1 (1)	
Missing	1	0	
Day 1 hCG (IU/l)	1889 [1315]	2224 [1366]	0.005
Day 4 hCG (IU/l)	1615 [1696.3], 172	3160 [1967], 113	<0.001
Ectopic pregnancy size, n (%)			
<2 cm	135 (71)	107 (80)	0.09
≥2 cm	54 (29)	26 (20)	

Data presented as mean (SD); median [IQR]; n (% excluding not stated/unknown), number where number different from group.

BMI: body mass index; hCG: human chorionic gonadotrophin; IVF: *in vitro* fertilization.

The rate of treatment success with single-dose methotrexate was 59% (n = 189/322). In the single-dose methotrexate treatment failure group, 65% (n = 86/133) had surgery only, 26% (n = 34/133) had a second-dose methotrexate only and 7% (n = 9/133) had surgery after a second-dose methotrexate. Four participants did not have hCG follow-up to point of resolution (<30 IU/l); of these, one woman was lost to follow-up, and three women withdrew from follow-up, with one reporting a negative urine pregnancy test. Allowing for additional methotrexate doses, the success rate of methotrexate was 69% (n = 222/322).

**Figure 1.**
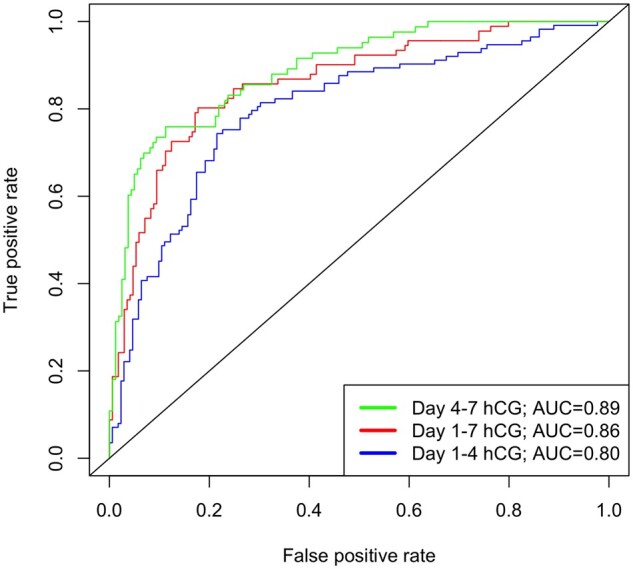
**Receiver operating characteristic (ROC) curve analysis of prediction of treatment success for Days 1–4, 1–7, and 4–7 serum hCG percentage changes.** The area under the curve (AUC) for each predictor is stated in the figure legend.

ROC curve analysis was performed (see Fig. 1) to evaluate Days 1–4, 1–7, and 4–7 serum hCG change and the AUC for each marker was calculated alongside an optimal classification threshold (see Table 2). Table 3 presents the probability of treatment success and positive likelihood ratios of hCG percentual change ranges across Days 1–4, 1–7, and 4–7 data. The sensitivity, specificity, PPV, NPV, likelihood ratio of a positive test and likelihood ratio of a negative test were calculated for optimal thresholds alongside previously investigated thresholds including a fall in hCG, a >15% fall and a >20% fall within each time interval assessed (see Table 4).

**Table 2.** Receiver operating characteristic (ROC) summary statistics.

Serum hCG interval	AUC (95% CI)	*P* value AUC	Optimal classification threshold of serum hCG change
Days 1–4	0.80 (0.74–0.85)	<0.001	18%
Days 1–7	0.86 (0.81–0.91)	<0.001	−5%
Days 4–7	0.89 (0.85–0.93)	<0.001	−15%

AUC: area under curve.

**Table 3.** Test performance characteristics presented across ranges of percentual change of serum hCG.

	Treatment success, n (%)	Treatment failure, n (%)	Likelihood ratio (95% CI)	Probability of treatment success, % (95% CI)
**Days 1–4 serum hCG change (n = 285)**				
>20% fall (n = 75)	64	11	3.82 (2.11–6.92)	85.3 (75.3–92.4)
0–20% fall (n = 42)	35	7	3.29 (1.51–7.14)	83.3 (68.6–93.0)
0–20% rise (n = 51)	36	15	1.58 (0.91–2.74)	70.6 (56.2–82.5)
20–40% rise (n = 41)	17	24	0.47 (0.26–0.83)	41.5 (26.3–57.9)
>40% rise (n = 76)	20	56	0.24 (0.15–0.37)	26.3 (16.9–37.7)
**Days 1–7 serum hCG change (n = 260)**				
>40% fall (n = 97)	88	9	5.27 (2.79–9.95)	90.7 (83.1–95.7)
20–40% fall (n = 40)	36	4	4.85 (1.78–13.19)	90.0 (76.3–97.2)
0–20% fall (n = 27)	17	10	0.92 (0.44–1.92)	63.0 (42.4–80.6)
0–20% rise (n = 31)	14	17	0.44 (0.23–0.86)	45.2 (27.3–64.0)
>20% rise (n = 65)	14	51	0.15 (0.09–0.25)	21.5 (12.3–33.5)
**Days 4–7 serum hCG change (n = 243)**				
>40% fall (n = 64)	62	2	16.08 (4.03–64.11)	96.9 (89.2–99.6)
20–40% fall (n = 73)	60	13	2.39 (1.40–4.10)	82.2 (71.5–90.2)
0–20% fall (n = 75)	34	41	0.43 (0.30–0.62)	45.3 (33.8–57.3)
Any rise (n = 31)	4	27	0.08 (0.03–0.21)	12.9 (3.6–29.8)

**Table 4.** Test performance characteristics.

	**Sensitivity** **% (95% CI)**	**Specificity** **% (95% CI)**	Likelihood ratio of positive test (95% CI)	Likelihood ratio of negative test (95% CI)	Positive predictive value % (95% CI)	Negative predictive value % (95% CI)
**Days 1–4 serum hCG change (n = 285)**						
<18% rise*(n = 164)	**78.5 (71.6–84.4)**	**74.3 (65.3–82.1)**	**3.01 (2.21–4.23)**	**0.29 (0.21–0.39)**	**82.3 (75.6–87.8)**	**69.4 (60.4–77.5)**
Any fall (n = 117)	57.6 (49.8–65.0)	84.1 (76.0–90.3)	3.61 (2.32–5.63)	0.51 (0.42–0.61)	84.6 (76.8–90.6)	56.5 (48.7–64.2)
>15% fall (n = 87)	43.6 (36.1–51.4)	89.4 (82.2–94.4)	4.11 (2.34–7.20)	0.63 (0.55–0.73)	86.2 (77.1–92.7)	51.0 (43.8–58.2)
>20% fall (n = 75)	37.2 (30.0–44.9)	90.3 (83.2–95.0)	3.82 (2.11–6.92)	0.70 (0.61–0.79)	85.3 (75.3–92.4)	48.6 (41.6–55.5)
**Days 1–7 serum hCG change (n = 260)**						
Any fall (n = 164)	83.4 (77.0–88.7)	74.7 (64.5–83.3)	3.30 (2.30–4.73)	0.22 (0.15–0.32)	86.0 (79.7–90.9)	70.8 (60.7–79.7)
>5% fall*(n = 156)	**81.7 (75.0–87.2)**	**80.2 (70.6–87.8)**	**4.13 (2.71–6.28)**	**0.23 (0.16–0.32)**	**88.5 (82.4–93.0)**	**70.2 (60.4–78.8)**
>15% fall (n = 143)	75.1 (67.9–81.5)	82.4 (73.0–89.6)	4.27 (2.72–6.72)	0.30 (0.23–0.40)	88.8 (82.5–93.5)	64.1 (54.7–72.8)
>20% fall (n = 137)	73.4 (66.0–79.9)	85.7 (76.8–92.2)	5.14 (3.08–8.57)	0.31 (0.24–0.40)	90.5 (84.3–94.9)	63.4 (54.3–71.9)
**Days 4–7 serum hCG change (n = 243)**						
Any fall (n = 212)	97.5 (93.7–99.3)	32.5 (22.6–43.7)	1.45 (1.24–1.68)	0.08 (0.03–0.21)	73.6 (67.1–79.4)	87.1 (70.2–96.4)
>15% fall* **(n = 162)**	**88.7 (82.8–93.2)**	**75.9 (65.3–84.6)**	**3.68 (2.50–5.42)**	**0.15 (0.09–0.23)**	**87.7 (81.6–92.3)**	**77.8 (67.2–86.3)**
>20% fall (n = 137)	76.2 (68.9–82.6)	81.9 (72.0–89.5)	4.22 (2.65–6.73)	0.29 (0.22–0.39)	89.1 (82.6–93.7)	64.2 (54.3–73.2)

* Optimal threshold (in bold type) identified via maximization of Youden's index. An <18% rise is inclusive of any fall in serum hCG.

ROC curve analysis showed that Days 1–4 hCG percentual change predicted single-dose methotrexate treatment success, with an AUC of 0.80 (95% CI 0.74–0.85) (Fig. 1). We identified an optimal Days 1–4 serum hCG threshold as an 18% rise. A fall in hCG between Day 1 and Day 4 was observed in 41% of participants with Days 1–4 hCG data (n = 117/285) and predicted treatment success with PPV 85%. A <18% rise (inclusive of any fall in hCG) was observed in 58% (n = 164/285) of participants with Days 1–4 hCG data and predicted treatment success with 82% PPV, 69% NPV, 79% sensitivity and 74% specificity (Table 4).

Among those with a falling Days 1–4 hCG, the median hCG change in the 24-h prior to treatment was a 4.2% rise (IQR 11.9; n = 95), establishing that the falling Days 1–4 hCG was not a product of a pre-existing downwards trend but more likely a consequence of the treatment effect.

Days 1–7 and Days 4–7 hCG had predictive value in determining treatment success with AUC of 0.86 (95% CI 0.81–0.91) and 0.89 (95% CI 0.85–0.93) respectively. Optimal thresholds for Days 1–7 and Days 4–7 hCG were identified as a 5% fall and a 15% fall respectively (Table 4). A >5% fall in Days 1–7 hCG was observed in 60% (n = 156/260) of participants and predicted treatment success with 89% PPV, 70% NPV, 82% sensitivity and 80% specificity. A Days 4–7 >15% hCG fall occurred in 67% (n = 162/243) of participants, predicting treatment success with 88% PPV, 78% NPV, 89% sensitivity and 76% specificity.

A subgroup analysis examined the predictive value of hCG trends in the methotrexate/gefitinib and methotrexate/placebo arms of the GEM3 study. In the methotrexate/placebo group, the rate of single-dose methotrexate treatment success was 58% (n = 92/159). In this group, ROC curve analysis was used to calculate the AUC for each marker. The AUC was 0.78 (95% CI 0.70–0.86), 0.84 (95% CI 0.77–0.92) and 0.91 (95% CI 0.86–0.97) for Days 1–4, 1–7, and 4–7 serum hCG change respectively (see Supplementary Fig. S1). To compare hCG predictors (Days 1–4, 1–7, and 4–7 serum hCG change) of treatment success in methotrexate/gefitinib and methotrexate/placebo groups, the AUC for each predictor in each group was compared between the groups. There was no evidence of statically significant differences in the AUC between the methotrexate/gefitinib and methotrexate/placebo groups for Days 1–4 (*P* = 0.48), Days 1–7 (*P* = 0.51), and Days 4–7 (*P* = 0.29) serum hCG changes.

## Discussion

To our knowledge, this is the largest prospective study assessing Days 1–4 serum hCG change as a predictor of single-dose methotrexate treatment success in tubal ectopic pregnancy. In our cohort, a fall in Days 1–4 serum hCG indicated an 85% (CI 76.8–90.6) likelihood of treatment success. Additionally, a <18% rise in Days 1–4 serum hCG signifies an 82% likelihood of treatment success, associated with high sensitivity and specificity. These findings support previous studies showing the predictive value of Days 1–4 hCG change (Agostini *et al.*, 2007; Nguyen *et al.*, 2010; Skubisz *et al.*, 2013; Wong *et al.*, 2018; Levin *et al.*, 2019; Goh *et al.*, 2020).

This study benefits from high-quality, carefully-phenotyped, prospectively collected data from a large multicentre clinical trial (GEM3) (Horne *et al.*, 2023). We analysed data from both arms of the trial (methotrexate/placebo and methotrexate/gefitinib) based on the GEM3 finding that gefitinib was non-superior to placebo and the lack of any significant differences in the AUC between subgroups for each hCG marker assessed.

Women with tubal ectopic pregnancy and hCG <1000 IU/l can be managed highly effectively with methotrexate or expectant management (Mavrelos *et al.*, 2013; van Mello *et al.*, 2013) and methotrexate is not recommended for those with hCG >5000 IU/l due to unacceptably high failure rates (Elson *et al.*, 2016). Reflecting this, our cohort included individuals with an immediate pre-treatment serum hCG of ≥1000 and ≤5000 IU/l. To date, no other studies assessing hCG change include a minimum and maximum hCG inclusion criterion, resulting usually in a lower Day 1 hCG in other cohorts compared to that presented herein (Agostini *et al.*, 2007; Kirk *et al.*, 2007; Nguyen *et al.*, 2010; Levin *et al.*, 2019). This should be considered where direct comparisons with other studies are made. Additionally, this study is limited to single-dose methotrexate treatment success in tubal ectopic pregnancy only, and we would caution extrapolation of our findings to non-tubal ectopic pregnancy or choriocarcinoma.

We have presented Days 1–4 serum hCG change alongside serum hCG changes reliant on Day 7 data (Days 1–7 and Days 4–7), although we caution their direct comparison for two key reasons. Firstly, they represent different timepoints; Day 4 and Day 7 hCG are not contemporaneously available to inform clinical judgement or patient counselling. A compromise in predictive value may be worthwhile if the information could be available 3 days earlier. Secondly, the effect of intervention bias on Days 4–7 serum hCG change is important to consider. Current guidance from both the Royal College of Obstetricians and Gynaecologists (Elson *et al.*, 2016) and the American College of Obstetricians and Gynecologists (2018) recommend that those who have a ≤15% reduction in Days 4–7 can be considered highly likely to fail treatment and should be managed with additional methotrexate or surgery. We would therefore expect clinicians to follow these recommendations, consequently inflating the observed predictive value of Days 4–7 hCG change in our cohort (i.e. the change in hCG between Days 1–4 will not influence clinician decision making while the Days 4–7 change might). Additionally, due to intervention bias it is difficult to ascertain whether the 15% fall in Days 4–7 hCG we identified reflects implementation of this clinical guidance as opposed to the intrinsic predictive value of the change in hCG itself.

Any fall in Days 1–4 serum hCG signifies an 85% likelihood of treatment success for women with tubal ectopic pregnancy and hCG of ≥1000 and ≤5000 IU/l managed with single-dose methotrexate. Similarly, a modest (<18%) Days 1–4 serum hCG rise signifies an 82% likelihood of single-dose methotrexate treatment success with improved sensitivity. We therefore recommend that women are counselled at Day 4 in accordance with their Days 1–4 hCG change (see Table 5). Those who see a fall or only a modest (<18%) rise in hCG can receive early reassurance that treatment for their life-threatening condition will likely be successful and that the likelihood of single-dose methotrexate treatment success has improved by 44% and 40% respectively compared to when it was administered.

**Table 5.** Implications for clinical practice.

Implications for clinical practice
We recommend women with tubal ectopic pregnancy managed with methotrexate have a serum hCG sampled on Day 4 post-methotrexate administration We recommend women are counselled on Day 4 post-methotrexate administration in light of their Days 1–4 serum hCG change Women with a fall or modest (<18%) rise in Days 1–4 hCG can be provided with early reassurance that their treatment will likely be effective Any fall in Days 1–4 serum hCG signifies an 85% likelihood of treatment success for women with tubal ectopic pregnancy and hCG of ≥1000 and ≤5000 IU/l managed with single-dose methotrexate A modest (<18%) rise in Days 1–4 hCG signifies an 82% likelihood of single-dose methotrexate treatment success for women with tubal ectopic pregnancy and hCG of ≥1000 and ≤5000 IU/l managed with single-dose methotrexate

## Supplementary Material

dead089_Supplementary_Figure_S1

## Data Availability

Data in this study are derived from the GEM3 trial (Horne *et al.*, 2023). Please see Horne *et al.* (2023) for data requests.
