# Supplementary material for: Early (Days 1–4) post-treatment serum hCG level changes predict single-dose methotrexate treatment success in tubal ectopic pregnancy
Source: Hum Reprod. 2023 May 13;38(7):1261–7. doi: 10.1093/humrep/dead089 (PMC10320483; doi:10.1093/humrep/dead089)
Supplement: dead089_Supplementary_Figure_S1 [file dead089_supplementary_figure_s1.pdf]

OXFORD
human reproduction
Human Reproduction, 2023, 1–1
https://doi.org/10.1093/humrep/dead089
Supplementary Data

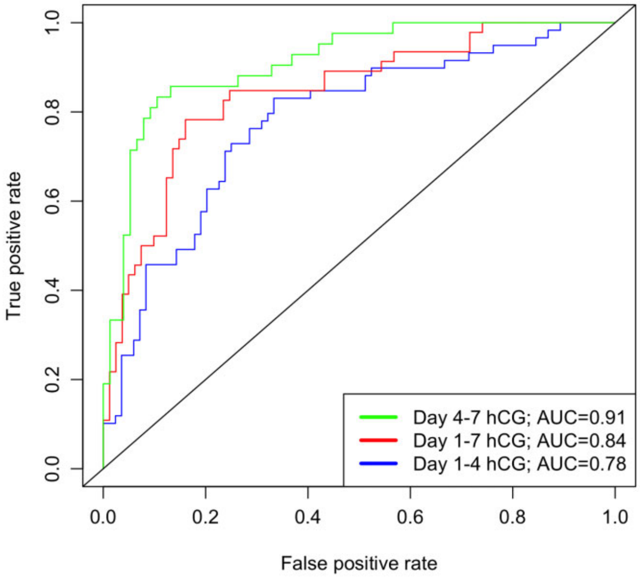

Supplementary Figure S1. Receiver operating characteristic (ROC) curve analysis of prediction of treatment success for Days 1–4, 1–7, and 4–7 serum hCG percentage changes in the methotrexate/placebo group. The area under the curve (AUC) for each predictor is stated in the figure legend.
